# Acteoside Counteracts Interleukin-1*β*-Induced Catabolic Processes through the Modulation of Mitogen-Activated Protein Kinases and the NF*κ*B Cellular Signaling Pathway

**DOI:** 10.1155/2021/8684725

**Published:** 2021-03-24

**Authors:** HyangI Lim, Do Kyung Kim, Tae-Hyeon Kim, Kyeong-Rok Kang, Jeong-Yeon Seo, Seung Sik Cho, Younghee Yun, Ye-yong Choi, Jungtae Leem, Hyoun-Woo Kim, Geon-Ung Jo, Chan-Jin Oh, Deuk-Sil Oh, Hong-Sung Chun, Jae-Sung Kim

**Affiliations:** ^1^Institute of Dental Science, Chosun University, Gwangju 61452, Republic of Korea; ^2^Departments of Biomedical Science, Chosun University, Gwangju 61452, Republic of Korea; ^3^Department of Biomedicine, Health & Life Convergence Sciences, BK21 Four, College of Pharmacy, Mokpo National University, Jeonnam 58554, Republic of Korea; ^4^Chung-Yeon Medical Institute, Gwangju 61949, Republic of Korea; ^5^Research and Development Institute, CY Pharma Co., Seoul 06224, Republic of Korea; ^6^Jeollanamdo Forest Resources Institute, Naju, Jeollanamdo, 58213, Republic of Korea

## Abstract

Osteoarthritis (OA) is the most common degenerative joint disease with chronic joint pain caused by progressive degeneration of articular cartilage at synovial joints. Acteoside, a caffeoylphenylethanoid glycoside, has various biological activities such as antimicrobial, anti-inflammatory, anticancer, antioxidative, cytoprotective, and neuroprotective effect. Further, oral administration of acteoside at high dosage does not cause genotoxicity. Therefore, the aim of present study is to verify the anticatabolic effects of acteoside against osteoarthritis and its anticatabolic signaling pathway. Acteoside did not decrease the viabilities of mouse fibroblast L929 cells used as normal cells and primary rat chondrocytes. Acteoside counteracted the IL-1*β*-induced proteoglycan loss in the chondrocytes and articular cartilage through suppressing the expression and activation of cartilage-degrading enzyme such as matrix metalloproteinase- (MMP-) 13, MMP-1, and MMP-3. Furthermore, acteoside suppressed the expression of inflammatory mediators such as inducible nitric oxide synthase, cyclooxygenase-2, nitric oxide, and prostaglandin E_2_ in the primary rat chondrocytes treated with IL-1*β*. Subsequently, the expression of proinflammatory cytokines was decreased by acteoside in the primary rat chondrocytes treated with IL-1*β*. Moreover, acteoside suppressed not only the phosphorylation of mitogen-activated protein kinases in primary rat chondrocytes treated with IL-1*β* but also the translocation of NF*κ*B from the cytosol to the nucleus through suppression of its phosphorylation. Oral administration of 5 and 10 mg/kg acteoside attenuated the progressive degeneration of articular cartilage in the osteoarthritic mouse model generated by destabilization of the medial meniscus. Our findings indicate that acteoside is a promising potential anticatabolic agent or supplement to attenuate or prevent progressive degeneration of articular cartilage.

## 1. Introduction

Osteoarthritis (OA) is the most common degenerative joint disease with chronic joint pain caused by progressive degeneration of articular cartilage at synovial joints [1]. Due to the increase in life expectancy, the prevalence of OA with loss of mobility and chronic joint pain caused by progressive degeneration of articular cartilage at synovial joints is estimated to be 18% and 9.6% in women after menopause and in men, respectively [2]. Although the worldwide prevalence of OA increases annually, the pathophysiological etiology of OA is still unknown. It may be caused by very complex and multifactorial risk factors such as aging, gender, genetic inheritance, traumatic joint injury, and severe mechanical joint load. Furthermore, the neuropathological relationships between progressive degeneration of articular cartilage and development of chronic joint pain are unknown [3]. Hence, the goal of clinical management for patients with OA is the maintenance of body mobility and mechanical joint function through relief from chronic joint pain, using pharmacological and nonpharmacological approaches and joint replacement surgery. The demand for development of effective intervention or supplementation, with long-term biological safety, to prevent or attenuate OA to maintain life quality through maintenance of mechanical joint function in the elderly population is increasing.

**Figure 1:**
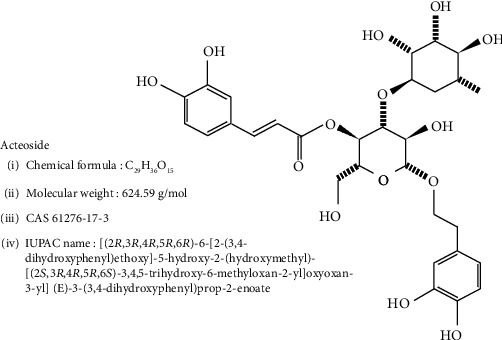
Chemical structure and information of acteoside.

As shown in Figure 1, acteoside (CAS No. 61276-17-3; C_29_H_36_O_15_) is a caffeoylphenylethanoid glycoside isolated from several herbal plants such as *Verbascum phlomoides* [4], *Buddleja globosa* [5], and *Plantago australis* [6]. Acteoside has various biological activities such as antimicrobial [5], anti-inflammatory [7], anticancer [8], antioxidative [9], cytoprotective [9], and neuroprotective effect [10]. Further, oral administration of acteoside at high dosage does not cause genotoxicity [11].

Hence, we hypothesized that acteoside with anti-inflammatory biological safety has anticatabolic effects associated with the protection of articular cartilage against progressive degeneration of articular cartilage through suppression of catabolic factors such as the proinflammatory cytokines, inflammatory mediators, and cartilage-degrading enzymes in synovial joints. Therefore, the aim of this study was to investigate the acteoside-induced anticatabolic effects and its cellular signaling pathway both *in vitro*, using primary chondrocytes isolated from the articular cartilage of rat knee joint, and *in vivo*, using an OA animal model generated by surgical destabilization of the median meniscus in the knee joint of mice.

## 2. Methods

### 2.1. Cell Culture

Primary rat chondrocytes were isolated from the articular cartilage of rat (5-day-old; Sprague–Dawley) knee joints, in accordance with the protocol (CIACUC2019-A0027) approved by the Institutional Animal Care and Use Committee of Chosun University, Gwangju, Republic of Korea. Isolated primary rat chondrocytes were maintained in Dulbecco's Modified Eagle's Medium/Nutrient Mixture F-12 (DMEM/F12) (Thermo Scientific, Rockford, IL, USA) supplemented with 10% fetal bovine serum (FBS) (Life Technologies, Grand Island, NY, USA), antibiotics (50 U/mL penicillin and 50 *μ*g/mL streptomycin), and 50 *μ*g/mL ascorbic acid. The normal mouse fibroblast L-929 cell line was purchased from American Type Culture Collection (ATCC). According to the instructions provided from ATCC, L-929 cells were cultured in the Eagle's minimum essential medium, containing 10% FBS, and were grown in a humidified incubator at 37°C with 5% CO_2_.

### 2.2. Cell Viability Assay

The dimethyl thiazolyl diphenyl tetrazolium salt (MTT) assay was performed to assess the viabilities of mouse fibroblast cell line L929 cells used as a normal cell and primary rat chondrocytes treated with acteoside. Briefly, L929 cells and primary rat chondrocytes were cultured at a cell density of 8 × 10^5^ cells/mL in culture plates for 24 h and then treated with 2.5, 5, 10, 25, 50, and 100 *μ*M acteoside for 24 h. After treatment with MTT solution, both L929 cells and chondrocytes were further cultured for 4 h. After incubation, the formed MTT crystals were suspended completely in dimethyl sulfoxide and measured for absorbance at 570 nm using a spectrometer (Epoch microplate spectrophotometer, BioTek®, Winooski, VT, USA) to assess cell viability.

### 2.3. Cell Live/Dead Assay

Cell survival was performed using Cell Live/Dead assay kit (Molecular Probes, Carlsbad, CA, USA), which is composed of green calcein AM for labeling live cells (with green fluorescence) and ethidium homodimer-1 for labeling dead cells (with red fluorescence). Briefly, both L929 cells and primary rat chondrocytes were cultured at a cell density of 8 × 10^5^ cells/mL on chamber slides (Nunc® Lab-Tek® Chamber Slide™ system; Sigma-Aldrich; Merck KGaA) for 24 h and then treated with 50 and 100 *μ*M acteoside for 24 h. After cultivation, cell survival assay was performed according to the manufacturer's instruction. Thereafter, stained cells were imaged using a fluorescence microscope (Eclipse TE200; Nikon Instruments, Melville, NY).

### 2.4. Dimethylmethylene Blue (DMMB) Assay

DMMB assay was performed to assess the alteration of proteoglycan content in primary rat chondrocytes treated with acteoside for 21 days in the presence or absence of IL-1*β*. To maintain the characteristics of primary rat chondrocytes for 21 days, primary rat chondrocytes (2 × 10^6^ cells) were suspended in 1 mL of 1.2% alginate and then encapsulated by dripping the cell/alginate suspension to a solution of 105 mM CaCl_2_. The primary rat chondrocytes encapsulated in alginate were cultured for 24 h in DMEM/F12 (containing 10% FBS, 50 U/mL penicillin, 50 *μ*g/mL streptomycin, and 50 *μ*g/mL ascorbic acid) and then adapted for 24 h in DMEM/F12 containing 1% mini-insulin–transferrin–selenium (mini-ITS) and 50 *μ*g/mL ascorbic acid. Subsequently, the chondrocytes were treated with 50 or 100 *μ*M acteoside in the presence or absence of 1 ng/mL IL-1*β* for 21 days. At day 21, the primary rat chondrocytes were collected for assessment of proteoglycan content using the DMMB assay, as described previously [12]. In addition, to quantify proteoglycan content per cell and assess the proliferation of primary rat chondrocytes, cell numbers were measured by DNA assay using PicoGreen (Molecular Probes, Carlsbad, CA), according to the manufacturer's instructions.

### 2.5. Ex Vivo Organ Culture of Rat Articular Cartilage Tissues

Articular cartilage tissues were isolated from the knee joints of 5-day-old Sprague–Dawley rats and then cultured in DMEM/F12 supplemented with 10% FBS. Next, the articular cartilage samples were treated with 100 *μ*M acteoside in the presence or absence of 10 ng/mL IL-1*β* for 7 days. At the end of the culture period, the samples were collected and fixed in 4% paraformaldehyde for 72 h for histological analysis.

### 2.6. Histological Analysis

Histological analysis using safranin-O and fast green staining was performed to verify proteoglycan loss in the articular cartilage treated with 100 *μ*M acteoside in the presence or absence of 10 ng/mL IL-1*β* for 7 days. Briefly, fixed articular cartilage samples were decalcified in ethylenediaminetetraacetic acid and then embedded in paraffin. Thereafter, the prepared paraffin blocks containing articular cartilage were serially sliced to 5 *μ*m thickness and placed on slides. Safranin-O and fast green staining was subsequently performed to assess proteoglycan loss in the articular cartilage ground substance. In addition, hematoxylin and eosin staining was performed to observe the general morphology of the articular cartilage.

### 2.7. Western Blotting

Western blotting was performed to investigate the expression of catabolic factors including MMP-13, MMP-1, MMP-3, inducible nitric oxide synthase (iNOS), and cyclooxygenase-2 (COX-2) and the alteration of cellular signaling molecules such as mitogen-activated protein kinases and nuclear factor-kappa B (NF*κ*B). Briefly, rat primary chondrocytes were treated with 50 or 100 *μ*M acteoside in the presence or absence of 10 ng/mL IL-1*β* for 24 h. Thereafter, rat primary chondrocytes were harvested by centrifugation and were lysed using lysis buffer (Cell Signaling Technology, Danvers, MA, USA) according to the manufacturer's instructions. In addition, to verify the nuclear translocation of NF*κ*B, rat primary chondrocytes were treated with 50 or 100 *μ*M acteoside in the presence or absence of 10 ng/mL IL-1*β* for 24 h. Thereafter, cytosolic and nuclear fractions were extracted using NE-PER™ Nuclear and Cytoplasmic extraction reagents (Thermo Scientific, Rockford, IL, USA) according to the manufacturer's instructions. The concentration of total protein extracted from primary rat chondrocytes was determined using a bicinchoninic acid protein assay kit (Thermo Scientific, Rockford, IL, USA) according to the manufacturer's instructions. In addition, the conditioned medium was collected to detect the levels of cartilage-degrading enzymes secreted from chondrocytes. Equal amounts of protein and conditioned medium were electrophoresed on sodium dodecyl sulfate-polyacrylamide gel electrophoresis (SDS-PAGE) and then transferred onto nitrocellulose membranes. Thereafter, western blotting was performed using targeted primary antibodies against MMP-13, MMP-1, MMP-3, iNOS, COX-2, phospho-ERK1/2, total-ERK1/2, phospho-p38, total-p38, phospho-JNK, total JNK, phospho-NF*κ*B, total NF*κ*B, *β*-actin, and lamin B. Immunoreactive bands were visualized using an enhanced chemiluminescence system (Thermo Scientific, Rockford, IL, USA) according to the manufacturer's instruction and then imaged by a Microchemi device (DNR Bioimaging Systems, Jerusalem, Israel).

### 2.8. Quantitative Polymerase Chain Reaction (qPCR) and Quantitative Real-Time PCR (qRT-PCR)

Primary rat chondrocytes were treated with 50 or 100 *μ*M acteoside in the presence or absence of 10 ng/mL IL-1*β* for 24 h. Thereafter, total RNA was isolated from the primary rat chondrocytes using TRIzol reagent (Invitrogen, Carlsbad, CA, USA) according to the manufacturer's instructions. Total RNA concentration was measured using a NanoDrop 2000 (Thermo Scientific, Rockford, IL, USA). To synthesize cDNA, 1 *μ*g RNA was reverse transcribed using a ThermoScript reverse transcription-PCR system (Invitrogen, Carlsbad, CA, USA) according to the manufacturer's instructions. qPCR of cDNA was performed using 2× TOPsimple™ DyeMIX-*n*Taq (Enzynomics, Seoul, Republic of Korea) and specific primers on a TaKaRa PCR Thermal Cycler Device (TaKaRa Bio Inc., Shiga, Japan). Thereafter, the PCR products were electrophoresed on an agarose gel to determine the expression levels of target genes. Glyceraldehyde 3-phosphate dehydrogenase (GAPDH) was used as an endogenous control. In addition, for qRT-PCR, cDNA was amplified using an Eco™ Real-Time PCR system (illumine Inc., San Diego, CA, USA). *β*-Actin was used as an endogenous control. The sequences of the primers used in the qPCR and qRT-PCR are summarized in Tables 1 and 2, respectively.

### 2.9. Gelatin Zymography

Gelatin zymography was performed to assess the activation of MMPs in primary rat chondrocytes. Briefly, primary rat chondrocytes were treated with 50 or 100 *μ*M acteoside in the presence or absence of 10 ng/mL IL-1*β* for 24 h. Thereafter, an equal volume of conditioned medium was electrophoresed on a 10% polyacrylamide gel copolymerized with 0.2% (1 mg/mL) porcine skin gelatin. After electrophoresis, the gel was incubated in zymogram renaturing buffer (50 mM Tris–HCl (pH 7.6), 10 mM CaCl_2_, 50 mM NaCl, and 0.05% Brij-35) at 37°C for 72 h. After renaturation of MMPs, the gel was stained with 0.1% Coomassie Brilliant Blue R250. Gelatinolytic bands were revealed as clear bands on a background uniformly stained light blue and then imaged using a digital camera.

### 2.10. Measurement of Nitric Oxide (NO)

Primary rat chondrocytes were treated with 50 or 100 *μ*M acteoside in the presence or absence of 10 ng/mL IL-1*β* for 24 h. Thereafter, 50 *μ*L of the conditioned medium was reacted with 50 *μ*L each of sulfanilamide and N-1-napthylethylenediamine dihydrochloride. Absorbance was then measured at 540 nm wavelength using a spectrophotometer (Epoch Spectrophotometer, BioTek, Winooski, VT, USA).

### 2.11. Prostaglandin E_2_ (PGE_2_) Assay

Primary rat chondrocytes were treated with 50 or 100 *μ*M acteoside in the presence or absence of 10 ng/mL IL-1*β* for 24 h. Thereafter, PGE_2_ concentration was measured using a PGE_2_ parameter assay kit (R&D Systems Inc., Minneapolis, MN, USA) according to the manufacturer's instructions.

### 2.12. Cytokine Array

Primary rat chondrocytes were treated with 50 *μ*M acteoside in the presence or absence of 10 ng/mL IL-1*β* for 24 h. Thereafter, total proteins were extracted and quantified as previously described [13]. Next, cytokine array was performed to investigate alteration in cytokine production, according the manufacturer's instructions (RayBiotech, Inc., Norcross, GA, USA).

### 2.13. Nuclear Translocation Assay

Primary rat chondrocytes were treated with 50 and 100 *μ*g/mL acteoside in the presence of 10 ng/mL IL-1*β*. After 30 min, primary rat chondrocytes were fixed with 1% paraformaldehyde, permeabilized in 0.2% Triton X-100, and extensively washed with phosphate-buffered saline. Nonspecific signals were blocked using normal goat serum. After multiple washes, the chondrocytes were incubated with rabbit anti-NF*κ*B antibodies followed by incubation with FITC-conjugated goat anti-rabbit IgG (Thermo Fisher Scientific, Waltham, MA, USA) overnight at 4°C. Thereafter, stained cells were imaged using a laser confocal scanning microscope system (Leica Microsystems, Wetzlar, Germany) at the Gwangju branch of Korea Basic Science Institute (Gwangju, Republic of Korea).

### 2.14. Generation of Osteoarthritic Animals

To generate osteoarthritic animals, the medial meniscus (DMM) was surgically destabilized in the knee joints of BALB/c mice (average body weight 19.3 ± 0.5 g) in accordance with IACUC guidelines (CIACUC2019-A0029). The OA-induced animals were treated orally with 5 and 10 mg/kg acteoside resolved in 5% ethanol (experimental group; *n* = 5) or vehicle (5% ethanol) (DMM group; *n* = 5) every other day for 8 weeks. At the end of the culture period, knee joints were dissected and fixed using 5% paraformaldehyde for 7 days to perform histological assessments. After safranin-O and fast green staining, imaged tissues of articular cartilage were examined in accordance with the Mankin grade [14, 15].

### 2.15. Statistical Analysis

The experimental data are presented as the mean ± standard deviation and were compared using analysis of variance, followed by post hoc multiple comparison (Tukey's test) using SPSS software version 25 (IBM Corp.) All the data, except the animal study, were obtained from three independent experiments.

## 3. Results

### 3.1. Acteoside Does Not Affect L929 Cell and Primary Rat Chondrocyte Viability

**Figure 2:**
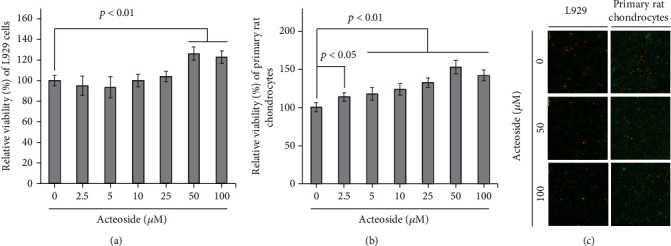
Acteoside does not affect L929 mouse fibroblast cell and primary rat chondrocyte viability. (a) Acteoside did not affect the viability of L929 mouse fibroblast cells. (b) Acteoside did not decrease the viability of primary rat chondrocytes. Mouse fibroblast cell line L929 used as normal cells and primary rat chondrocytes were treated with 2.5, 5, 10, 25, 50, and 100 *μ*M acteoside for 24 h. Thereafter, MTT assay was performed to assess the cytotoxicity of acteoside in L929 cells and primary rat chondrocytes. (c) Acteoside did not increase the cytotoxicity in both L929 and primary rat chondrocytes. To perform the Cell Live/Dead assay, L929 and primary rat chondrocyte were treated with 50 and 100 *μ*M acteoside for 24 h. Thereafter, Cell Live/Dead assay was performed. Stained cells were imaged using a fluorescence microscope (Eclipse TE200; Nikon Instruments, Melville, NY).

The mouse fibroblast cell line L929 used as normal cells was treated with 2.5, 5, 10, 25, 50, and 100 *μ*M acteoside for 24 h. Thereafter, the MTT assay was performed to assess the cytotoxicity of acteoside on L929 cells. As shown in Figure 2(a), relative viabilities of L929 cells were determined to be 94.8 ± 8%, 93.6 ± 7%, 100 ± 5%, 103.9 ± 5%, 126.1 ± 8%, and 122.7 ± 4% at 2.5, 5, 10, 25, 50, and 100 *μ*M acteoside, respectively, compared with control (100.02 ± 3%). Furthermore, to verify the cytotoxicity of acteoside on primary rat chondrocytes, the MTT assay was performed as shown in Figure 2(b). The viabilities of primary rat chondrocytes treated with 2.5, 5, 10, 25, 50, and 100 *μ*M acteoside were determined as 114 ± 4%, 117.8 ± 6%, 123.9 ± 5%, 132.6 ± 4%, 153.1 ± 7%, and 142.1 ± 6%, respectively, compared with control (100.4 ± 5%). Furthermore, to confirm the effect of acteoside on the viability of both L929 cells and primary rat chondrocytes, Cell Live/Dead assay was performed as shown in Figure 2(c). The number of dead cells stained as red fluorescence did not increase for both L929 cells and primary rat chondrocytes treated with 50 and 100 *μ*M acteoside for 24 h. These data consistently demonstrated that defined dosage of acteoside did not affect the viability of L929 cells and primary rat chondrocytes. Thus, 50 *μ*M acteoside and 100 *μ*M acteoside, which are nontoxic doses in both L929 cells and primary rat chondrocytes, were used to verify its anticatabolic effects in *in vitro* studies using primary rat chondrocytes.

### 3.2. Acteoside Counteracts IL-1*β*-Induced Proteoglycan Loss in Primary Rat Chondrocytes

**Figure 3:**
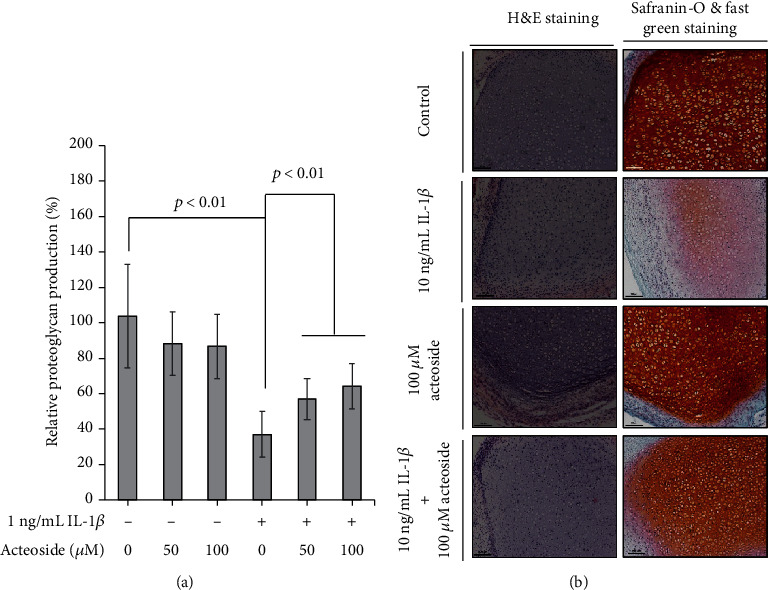
Acteoside counteracts IL-1*β*-induced proteoglycan loss in primary rat chondrocytes. (a) Acteoside rescued the proteoglycan production in the primary rat chondrocytes treated with IL-1*β*. Primary rat chondrocytes embedded in alginate were treated with 50 and 100 *μ*M acteoside in the presence or absence of 1 ng/mL IL-1*β* for 21 days. Thereafter, DMMB assay was performed to verify the alteration of proteoglycan contents. (b) Acteoside inhibits the IL-1*β*-induced proteoglycan loss in the articular cartilage dissected from rat knee joints. The tissues of articular cartilage were dissected from rat knee joint and were treated with 100 *μ*M acteoside in the presence or absence of 10 ng/mL IL-1*β* for 7 days. Thereafter, histological assessment using safranin-O and fast green staining was performed to verify the alteration of proteoglycan loss. Stained tissues were imaged using a microscope (Eclipse TE200; Nikon Instruments, Melville, NY).

Primary rat chondrocytes embedded in alginate beads were treated with 50 and 100 *μ*M acteoside in the presence or absence of 1 ng/mL IL-1*β* for 21 days. Thereafter, DMMB assay was performed to assess the alteration in proteoglycan content as shown in Figure 3(a). The relative proteoglycan contents were determined as 88.3 ± 18.1% and 86.8 ± 16.3% in the primary rat chondrocytes treated with 50 and 100 *μ*M acteoside, respectively, compared with control (103.8 ± 32.3%). Although the relative proteoglycan contents were decreased by acteoside, these results were not significant. However, the relative proteoglycan content significantly decreased by 37.1 ± 14.7% in the primary rat chondrocytes treated with 1 ng/mL IL-1*β*, but 50 and 100 *μ*M acteoside significantly reduced the proteoglycan content by 57 ± 12.4% and 64 ± 14.5%, respectively, in the presence of 1 ng/mL IL-1*β*. Subsequently, to verify whether acteoside suppresses the IL-1*β*-induced proteoglycan loss, articular cartilage dissected from rat knee joints was treated with 100 *μ*M acteoside in the presence or absence of 10 ng/mL IL-1*β* for 7 days. Thereafter, histological assessments using H&E staining and safranin-O and fast green staining were performed as shown in Figure 3(b). Morphological alteration was not observed using H&E staining; however, safranin-O and fast green staining revealed that the proteoglycan stained as red color did not alter in the articular cartilages treated with 100 *μ*M acteoside compared with that in control. However, severe proteoglycan loss was induced by 10 ng/mLIL-1*β* in the articular cartilage, and 100 *μ*M acteoside significantly suppressed the proteoglycan loss in the articular cartilage treated with 10 ng/mL IL-1*β*. Collectively, these data consistently show that acteoside has an anticatabolic effect that retards the degeneration of articular cartilage through counteracting IL-1*β*-induced proteoglycan loss.

### 3.3. Acteoside Has an Anticatabolic Effect That Suppresses MMP Expression and Activation in Primary Rat Chondrocytes Treated with IL-1*β*

**Figure 4:**
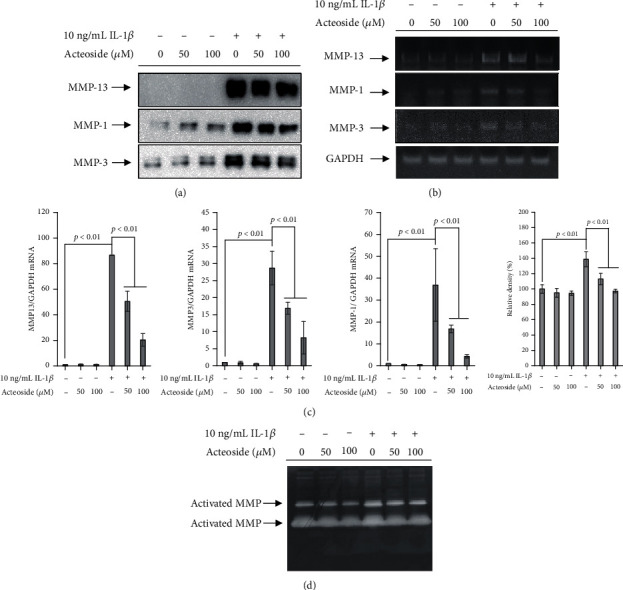
Acteoside has an anticatabolic effect that suppresses MMP expression and activation in primary rat chondrocytes treated with IL-1*β*. (a–c) Acteoside suppressed the expression of cartilage degrading enzymes such as MMP-13, MMP-1, and MMP-3 in the primary rat chondrocytes treated with IL-1*β*. Primary rat chondrocytes were treated with 50 and 100 *μ*M acteoside in the presence or absence of 10 ng/mL IL-1*β* for 24 h. Thereafter, total proteins and total RNA were extracted to perform western blot (a), quantitative PCR (b), and quantitative real-time PCR (c). (d) Acteoside suppressed the activation of cartilage-degrading enzymes. Primary rat chondrocytes were treated with 50 and 100 *μ*M acteoside in the presence or absence of 10 ng/mL IL-1*β* for 24 h. Thereafter, zymography was performed to verify the alteration of activated MMPs.

To investigate whether acteoside-induced anticatabolic effect is associated with the suppression of MMP expression and activation, primary rat chondrocytes were treated with 50 and 100 *μ*M acteoside in the presence or absence of 10 ng/mL IL-1*β* for 24 h. Thereafter, the alterations in MMPs were investigated. As shown in Figure 4(a), although the expression of cartilage-degrading enzymes such as MMP-13, MMP-1, and MMP-3 was significantly increased in the conditioned media of primary rat chondrocytes treated with 10 ng/mL IL-1*β*, it was decreased by acteoside in a dose-dependent manner. Furthermore, results of both qPCR (Figure 4(b)) and qRT-PCR (Figure 4(c)) revealed that IL-1*β* significantly increased the mRNA levels of MMPs such as MMP-13, MMP-1, and MMP-3 in the primary rat chondrocytes. However, they decreased dose-dependently in the primary rat chondrocytes treated with 50 and 100 *μ*M acteoside. Moreover, 50 and 100 *μ*M acteoside effectively suppressed the activation of MMPs in the rat primary chondrocytes treated with 10 ng/mL IL-1*β* (Figure 4(d)). Taken together, these data consistently indicate that acteoside has an anticatabolic effect that suppresses the expression and activation of cartilage-degrading enzymes.

### 3.4. Acteoside Suppresses the Expression and Production of IL-1*β*-Induced Catabolic Inflammatory Mediators and Proinflammatory Cytokines in Primary Rat Chondrocytes

**Figure 5:**
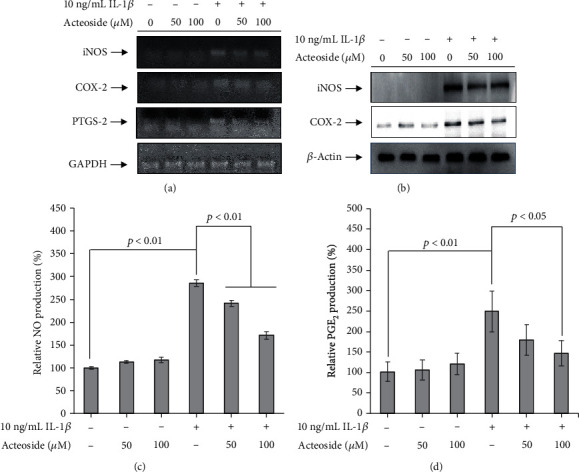
Acteoside suppresses the expression and production of IL-1*β*-induced catabolic inflammatory mediators in primary rat chondrocytes. (a, b) Acteoside suppressed the expression of inflammatory mediators in the primary rat chondrocytes treated with IL-1*β*. Primary rat chondrocytes were treated with 50 and 100 *μ*M acteoside in the presence or absence of 10 ng/mL IL-1*β* for 24 h. Thereafter, total proteins and total RNA were extracted to perform quantitative PCR (a) and western blot (b). (c, d) Acteoside suppressed the production of NO (c) and PGE_2_ (d) in the primary rat chondrocytes treated with IL-1*β*. Primary rat chondrocytes were treated with 50 and 100 *μ*M acteoside in the presence or absence of 10 ng/mL IL-1*β* for 24 h. Thereafter, the NO assay (c) and PGE_2_ assay (d) were performed on conditioned media.

**Figure 6:**
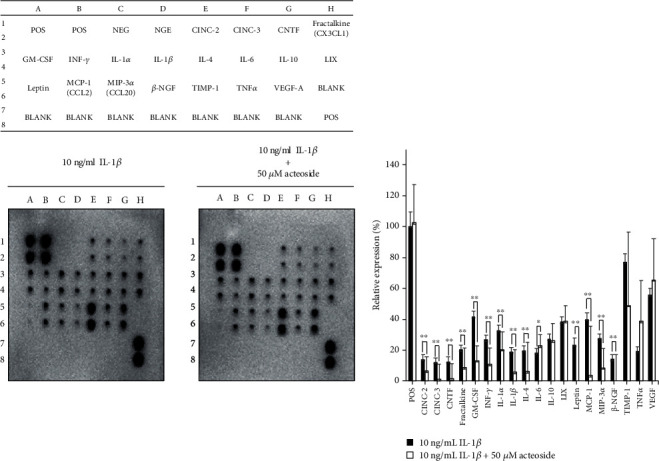
Acteoside suppressed the expression of IL-1*β*-induced catabolic proinflammatory cytokines, chemokines, and growth factors in primary rat chondrocytes. Primary rat chondrocytes were treated with 50 *μ*M acteoside in the presence or absence of 10 ng/mL IL-1*β* for 24 h. Total proteins were extracted, and the cytokine array was performed according the manufacturer's instructions.

To determine whether acteoside has a preventive effect against OA, primary rat chondrocytes were treated with 50 and 100 *μ*M acteoside in the presence or absence of 10 ng/mL IL-1*β* for 24 h. Thereafter, the alterations in inflammatory mediators, representative catabolic factors such as iNOS, COX-2, and PGE_2_, were investigated. The mRNA levels of iNOS, COX-2, and PTGS-2 were significantly increased by IL-1*β* in the primary rat chondrocytes. However, they decreased dose-dependently in the primary rat chondrocytes treated with acteoside (Figure 5(a)). Furthermore, acteoside not only suppressed the expression of iNOS and COX-2 in the primary rat chondrocytes treated with IL-1*β* (Figure 5(b)) but also significantly decreased the relative production of NO and PGE_2_ as shown in Figures 5(c) and 5(d), respectively. These data suggest that acteoside suppresses the expression of inflammatory mediator-induced proinflammatory cytokines that act as catabolic factors to induce the progressive degeneration of articular cartilage. Hence, to investigate the expressional alteration of proinflammatory cytokines by 50 *μ*M acteoside in the primary rat chondrocytes treated with 10 ng/mL IL-1*β*, cytokine array was performed as shown in Figure 6. Acteoside suppressed the expression of cytokine-induced neutrophil chemoattractant- (CINC-) 2, CINC-3, ciliary neurotrophic factor (CNTF), fractalkine (CX3CL1), IL-1*α*, IL-1*β*, leptin, monocyte chemoattractant protein-1 (MCP-1), macrophage inflammatory protein- (MIP-) 3*α*, and *β*-nerve growth factor (NGF) in the primary rat chondrocytes treated with IL-1*β* compared with IL-1*β* alone. Taken together, these data suggest consistently that acteoside prevents the progressive degeneration of articular cartilage through suppression of inflammatory mediators and proinflammatory cytokines against the IL-1*β*-induced catabolic effects in primary rat chondrocytes.

### 3.5. Acteoside Suppresses MAPK and NF*κ*B Phosphorylation in Primary Rat Chondrocytes Treated with IL-1*β*

**Figure 7:**
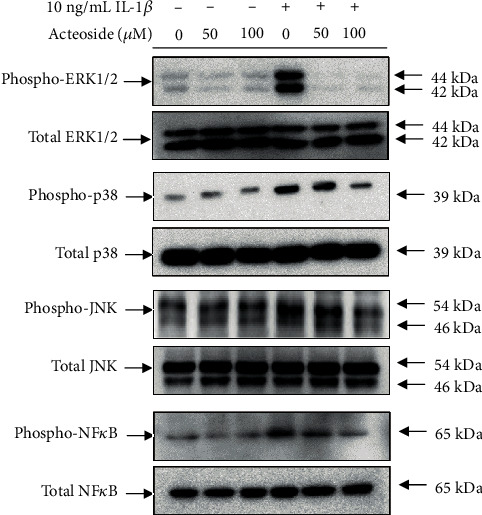
Acteoside suppresses MAPK and NF*κ*B phosphorylation in primary rat chondrocytes treated with IL-1*β*. Primary rat chondrocytes were treated with 50 and 100 *μ*M acteoside in the presence or absence of 10 ng/mL IL-1*β* for 24 h. Thereafter, total proteins were extracted to perform western blot using MAPK and NF*κ*B antibody.

To investigate the cellular signaling pathways associated with acteoside-induced anticatabolic effects against proinflammatory cytokine IL-1*β* alteration of MAPK and NF*κ*B, primary rat chondrocytes were treated with 50 and 100 *μ*M acteoside in the presence or absence of IL-1*β* for 24 h. Thereafter, total protein was extracted and electrophoresed on the SDS-PAGE gel to perform the western blot. As shown in Figure 7, MAPK such as ERK1/2, p38, and JNK were significantly phosphorylated in the primary rat chondrocytes treated with IL-1*β*, whereas 50 and 100 *μ*M acteoside did not significantly induce the phosphorylation of MAPK compared to the control in primary rat chondrocyte. However, 50 and 100 *μ*M acteoside dose-dependently suppressed the IL-1*β*-induced MAPK phosphorylation in primary rat chondrocytes. Furthermore, the phosphorylation of NF*κ*B in the primary rat chondrocytes treated with 10 ng/mL IL-1*β* was gradually decreased by acteoside in a dose-dependent manner. These data indicate that MAPK and NF*κ*B cellular signaling pathways are closely involved with the acteoside-induced anticatabolic effects against to IL-1*β* in primary rat chondrocytes.

### 3.6. Acteoside Suppresses Translocation of NF*κ*B from the Cytosol to the Nucleus through Suppression of IL-1*β*-Induced NF*κ*B Phosphorylation in Primary Rat Chondrocytes

**Figure 8:**
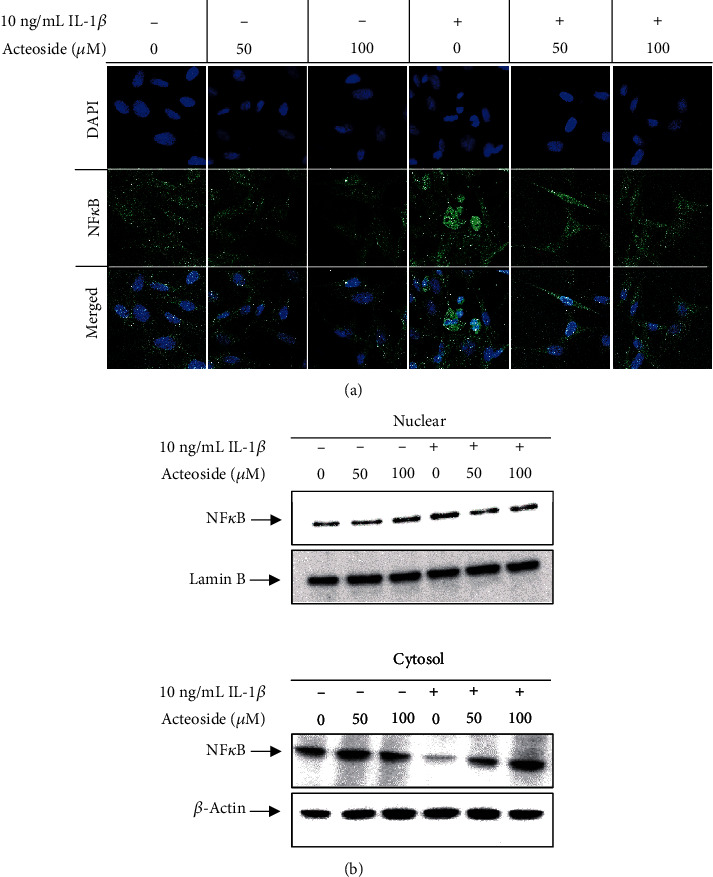
Acteoside suppresses translocation of NF*κ*B from the cytosol to the nucleus through suppression of IL-1*β*-induced NF*κ*B phosphorylation in primary rat chondrocytes. (a) Acteoside suppressed the nucleus translocation of NF*κ*B in the primary rat chondrocytes treated with IL-1*β*. Primary rat chondrocytes cultured on the chamber slide were treated with 50 and 100 *μ*M acteoside in the presence or absence of 10 ng/mL IL-1*β* for 24 h. Thereafter, the nucleus translocation was imaged using a laser confocal scanning microscope system (Leica Microsystems, Wetzlar, Germany). (b) The translocation of NF*κ*B from the cytosol to nucleus was suppressed by acteoside in primary rat chondrocytes treated with IL-1*β*. Primary rat chondrocytes were treated with 50 and 100 *μ*M acteoside in the presence or absence of 10 ng/mL IL-1*β* for 24 h. Thereafter, cytosolic and nucleus proteins were extracted and western blot was performed.

To investigate whether acteoside suppresses the translocation of NF*κ*B from the cytosol to the nucleus, primary rat chondrocytes were treated with 50 and 100 *μ*g/mL acteoside in the presence or absence of 10 ng/mL IL-1*β*. As shown in Figure 8(a), NF*κ*B was significantly translocated to the nucleus from the cytosol of the primary rat chondrocytes treated with 10 ng/mL IL-1*β*. However, it was significantly inhibited by acteoside in a dose-dependent manner. Furthermore, although the NF*κ*B level was increased in the nuclear fraction extracted from the primary rat chondrocytes treated with 10 ng/mL IL-1*β*, it was dose-dependently decreased by acteoside as shown in Figure 8(b). NF*κ*B level was decreased in the cytosolic fraction extracted from the primary rat chondrocytes treated with 10 ng/mL IL-1*β*, but it was dose-dependently increased by acteoside. Taken together, these data consistently indicate that acteoside-induced anticatabolic effects against IL-1*β* are involved in the suppression of translocation from the cytosol to the nucleus on the modulation of the NF*κ*B signaling pathway in primary rat chondrocytes.

### 3.7. Acteoside Attenuates Progressive Degeneration of Articular Cartilage in the Surgical DMM-Induced Knee Joint OA Animals

**Figure 9:**
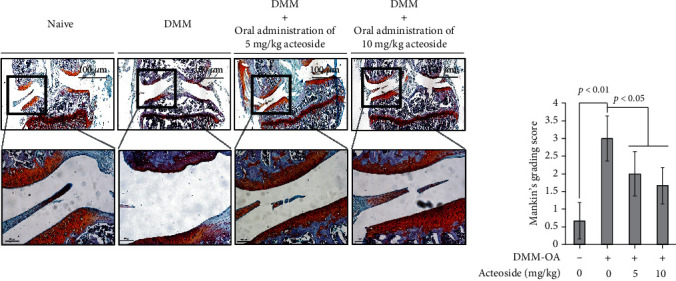
Acteoside attenuates progressive degeneration of articular cartilage in the surgical DMM-induced knee joint OA animals. (a) Progressive degeneration of articular cartilage was attenuated by the oral administration of acteoside in the surgical DMM-induced knee joint OA animals. OA animals were generated by the DMM surgery on the knee joint and were orally administrated 5 and 10 mg/kg acteoside resolved in 5% ethanol every other day for 8 weeks. At the end of day, knee joints were dissected, fixed, decalcified, embedded, and sliced to performed safranin-O and fast green staining. Tissues were imaged using a microscope (Eclipse TE200; Nikon Instruments, Melville, NY). (b) Acteoside counteracted the Mankin grading score in the surgical DMM-induced knee joint OA animals. After safranin-O and fast green staining, imaged tissues of articular cartilage were examined in accordance with the Mankin grade.

To elucidate the acteoside-induced anticatabolic effects *in vivo*, OA-induced animals generated by the surgical DMM performed on the knee joint of BALB/c mice were orally administrated 5 and 10 mg/kg acteoside resolved in 5% ethanol every other day for 8 weeks. Thereafter, knee joints were histologically assessed using safranin-O and fast green staining as shown in Figure 9. The proteoglycan loss and injury of articular cartilage surface were significantly increased in the knee joint dissected from DMM-induced OA animals. However, the oral administration of acteoside suppressed the proteoglycan loss and less injury of articular cartilage compared with vehicle only. Furthermore, Mankin's grading score was significantly increased in the OA animal group (*n* = 5, 3 ± 0.7) supplied vehicle only compared with Naïve (*n* = 5, 0.67 ± 0.5). However, the oral administration of 5 and 10 mg/kg acteoside into the OA animal group (*n* = 5) decreased the Mankin grading score by 2 ± 0.7 and 1.67 ± 0.5, respectively, compared with vehicle only. Taken together, these data indicate that the oral administration of acteoside attenuates the progressive degeneration of articular cartilage in synovial joint with catabolic conditions.

## 4. Discussion

The synovial (diarthrosis) joint is a complex anatomical structure consisting of several different types of tissues located at the potential space between bones to permit mobility and stability at the body through counteracting the different intensities of mechanical loading and control fine movements [16]. As the elderly population is increasing worldwide, OA is emerging as a degenerative disease associated with psychological and socioeconomic problems that are to be solved urgently [17]. Unfortunately, there are still no effective medications for OA; therefore, the prevention of articular cartilage degeneration is the most important to maintain the mechanical joint functions associated with the permission of body mobility and stability.

Generally, the synovial joint is composed of two bones to provide stability and support the muscle by ligament and tendons and is surrounded by a synovial fibrous joint capsule filled with synovial fluid to reduce friction between the articular cartilages located on the articular surfaces of the joined bones [16]. Especially, the extracellular matrix (ECM) of articular cartilage is composed mainly of type II collagen and proteoglycans that are synthesized and regulated by specialized cells called as chondrocytes. The homeostasis of articular cartilage is precisely balanced between anabolism (synthesis of ECM) and catabolism (degeneration of ECM) in synovial joints [18]. Generally, catabolic factors such as proinflammatory cytokines and inflammatory mediators induce the progressive degeneration of articular cartilage through the expression of cartilage-degrading enzymes such as matrix metalloproteinase (MMPs) and metalloproteinase with thrombospondin motifs (ADAMTs) from chondrocytes [18]. Hence, recent biochemical strategies to prevent or attenuate the progressive degeneration of articular cartilage have targeted the suppression of cartilage-degrading enzymes, proinflammatory cytokines, and inflammatory mediators based on the long-term biological safeties in synovial joints [19, 20]. Recent studies demonstrate that natural products, originating from herbal or oriental medicine, possess long-term biological safeties, anti-inflammatory, and antioxidative properties and may promote the joint health and managing OA through suppressing the release of proinflammatory cytokines [21].

Acteoside (called as verbascoside; C_29_H_36_O_15_) is a glycoside that is isolated from the flowers or leaves of many herbal plants such as *Scrophularia ningpoensis*, *Cistanche deserticola*, *Digitalis purpurea*, and *Osmanthus fragrans* [22, 23]. Recently, Henn et al. reported that the high concentration (100 *μ*g/mL) of acteoside isolated from the leaves of *Plantago australis* did not only show a less cytotoxicity in V79 Chinese hamster cells used as a normal cell but also did not have mutagenic or genotoxic activities and phototoxic properties [6]. Furthermore, Perucatti et al. have reported that *in vivo* cytogenetic test that is feeding 5 mg/kg acteoside to rabbit (*Oryctolagus cuniculus*) for 80 days revealed no toxicity with any other mutagenic activity, resulting in no cytotoxicity for the animals [24]. These studies suggest that acteoside is a bioactive material that can be used in both animal and human diets [6, 24]. As shown in Figure 2, similar with previous studies, 100 *μ*M (62.459 *μ*g/mL) acteoside did not affect the viabilities of mouse fibroblast cell line L929 used as a normal cell and primary rat chondrocytes in the present study. Hence, these data indicate that acteoside may have secured a potential biological safety and can be used as a supplement.

ECM, a large amount up to 98% of cartilage volume, is a highly organized network of hyaluronan, proteoglycans, and type II collagen [25]. Especially, proteoglycans are proteins glycosylated with sulfated glycosaminoglycan to form aggregating network that generate a static charge density to counteract compressive forces during the mechanical function of synovial joints [25]. Hence, the loss of proteoglycan in the articular cartilage of synovial joints leads to disability of mechanical joint function [25]. Degeneration of articular cartilage due to the loss of proteoglycan results in the imbalance between anabolic and catabolic process. Hence, recent biological strategies related with the regeneration of articular cartilage and the prevention or attenuation of progressive articular cartilage degeneration are considering the increase of anabolic process through the synthesis of major articular cartilage component such as proteoglycan and type II collagen and the increase of anticatabolic process against catabolic factors such as proinflammatory cytokines, inflammation mediators, and catabolic growth factors. As shown in Figure 3, acteoside did not only recover the proteoglycan content through the counteraction against proinflammatory cytokine IL-1*β*-induced proteoglycan depletion in the primary rat chondrocytes but also suppressed the proteoglycan loss in the articular cartilage tissues treated with IL-1*β* for 7 days. Taken together, these data indicate consistently that acteoside may protect or attenuate the progressive degeneration of articular cartilage through counteraction to proinflammatory cytokine-induced catabolic process in the articular cartilage of synovial joint.

Elevated cartilage-degrading enzymes including MMP-1, MMP-3, MMP-13, ADMATS-4, and ADAMTS-5 in the synovial fluid of patients with OA are the key enzymes responsible for the progressive degeneration of articular cartilage through degradation of collagen and ECM component [26, 27]. Hence, the inhibition of MMP expression and activation seems to be an attractive therapeutic strategy to prevent and attenuate the progressive degeneration of articular cartilage for maintaining the mechanical function of synovial joints [26]. In the present study, acteoside effectively suppressed the expression and activation of cartilage-degrading enzyme in the primary rat chondrocytes treated with proinflammatory cytokine IL-1*β* as shown in Figure 4. These data indicate that acteoside may attenuate the progressive degeneration of articular cartilage through suppressing the expression and activation of articular cartilage in the synovial joint with catabolic conditions.

The inflammatory mediators such as iNOS, NO, COX-2, and PGE_2_ are integral to OA pathogenesis [28]. Especially, proinflammatory cytokines such as IL-1*β* and TNF*α* upregulate the production of NO and PGE_2_ through the increase of iNOS and COX2, respectively, in the synovial joint with OA [29, 30]. Upregulated NO inhibits the synthesis of ECM component such as type II collagen and proteoglycan. Besides, increased PGE_2_ inhibits the proliferation of chondrocytes and reduces the synthesis of ECM [28]. Hence, suppression of inflammatory mediators may attenuate the progressive degeneration of articular cartilage through the inhibition of ECM reduction in the synovial joint with OA. In the present study, acteoside effectively suppressed the upregulation of inflammatory mediators as shown in Figure 5. These data indicate consistently that acteoside may attenuate the progressive degeneration of articular cartilage through the suppression of inflammatory mediators in the synovial joint with OA.

Moreover, the overexpression of proinflammatory cytokines by the inflamed synovium and chondrocytes is a major risk pathogenic factor in OA pathogenesis. Especially, the expression of proinflammatory cytokine is thought to be generated by the synovial membrane at the stage of OA initiation. Sequentially, upregulated proinflammatory cytokines activate chondrocytes to express their own expression and to synthesize the cartilage-degrading enzymes, chemokines, and inflammatory mediators [31]. Therefore, the suppression of proinflammatory cytokines can prevent OA and may attenuate the progressive degeneration of articular cartilage through the inhibition of other proinflammatory cytokines, inflammatory mediators, and cartilage-degrading enzymes. In the present study, acteoside suppressed the production of proinflammatory cytokines such as CINC-2, CINC-3, CNTF, fractalkine, IL-1*α*, IL-1*β*, leptin, MCP-1, MIP-3*α*, and *β*-NGF in primary rat chondrocytes treated with IL-1*β* compared with IL-1*β* alone, as shown in Figure 6.

Gouze et al. reported that CINC-2 was significantly increased in chondrocytes treated with IL-1*β* similar with our study [32]. However, a recent study showed that spinal processing of painful inputs is closely altered during OA pathogenesis [33]. With regard to joint pain, CINC-2 and CINC-3 were significantly upregulated in the spinal dorsal horn of OA animals generated by the intra-articular injection of monosodium iodoacetate into knee joint [34, 35]. Although the pathophysiological role of CINC-2 and CINC-3 in OA pathogenesis is still largely unknown, these studies indicate that the expression of CINC-2 and CINC-3 in the spinal dorsal horn under OA conditions may be closely associated with the development of joint pain during OA pathogenesis.

CNTF, which is a pluripotent neurotropic factor and is related with the cytokine family that includes IL-6, IL-11, leukemia inhibitory family, and oncostatin, binds and signals to maintain the bone homeostasis through the gp130 coreceptor subunit [36]. Although the biological function of CNTF is still largely unknown in OA, recent studies have shown that CNTF-gp130 signaling may be associated with the pathologic bone remodeling evident in rheumatoid arthritis (RA), periodontal disease, spondyarthropathies, and OA through regulating the differentiation and activity of osteoblast, osteoclast, and chondrocytes [36]. In addition, a recent study showed that *β*-NGF, a neurotrophic factor involved with the physiological regulation of neuronal cells, was upregulated in blood and synovial fluid in the patient with OA [37]. However, several studies have reported that the blockade of NGF reduces the OA pain [38–40]. Therefore, neurotropic factors including CNTF and NGF not only are considered a pathogenic risk factor of OA progression but also provide the neurological linkage between the progressive degeneration of articular cartilage and the development of chronic OA pain. Furthermore, it has been considered a therapeutic targeting molecule to reduce the chronic OA pain.

Fractalkine also known as chemokine CX3CL1 is exuberantly expressed in both adult human and rat articular chondrocytes treated with IL-1*β* [41, 42]. Recent studies have reported that fractalkine promotes the expression of MMP-3 through the CX3CR1, c-Raf, MEK, ERK, and NF*κ*B cellular signaling pathways in the synovial tissue obtained from the patients with OA [43]. Furthermore, the genomic-wide DNA methylation analysis in OA chondrocytes revealed that fractalkine gene was not only hypomethylated but also constantly correlated with its mRNA expression [44]. MCP-1, a member of chemokine family to induce the inflammation, trigger the chemotaxis and transendothelial migration of monocyte to inflammatory lesion. Recently, Xu et al., have reported that MCP-1 and chemokine (C-C motif) receptor 2 axis are involved with the degradation of articular cartilage through the expression of MMP-13 and the increase of OA chondrocyte apoptosis [45]. Furthermore, MIP-3*α* also called as a chemokine CCL20 is abundantly expressed in the articular cartilage of patients with OA and increases the progressive degeneration of articular cartilage through the expression of cartilage-degrading enzymes such as MMP-1 and MMP-3, inflammatory mediator such as PGE_2_, and proinflammatory cytokine IL-6 [46]. Hence, chemokines such as fractalkine, MCP-1, and MIP-3*α* have been also considered a pathophysiological risk factor to initiate the progression of OA.

Leptin is a peptide hormone belonging to adipokines, which are cytokines secreted by adipose tissue [47]. Recent studies have reported that the level of leptin is not only elevated significantly in the human body with obesity but also increased in the serum and synovial fluid collected from the patients with OA that is correlated with the severity of OA [48]. Hence, resent studies have suggested that the expressions of leptin and its receptor have been considered positively as a risk factor associated with the development of OA [49–51]. [52]

IL-1 family, including IL-1*α* and IL-1*β*, is considered the most key cytokine associated with the pathogenesis of OA that induces the inflammatory catabolic process combined with other catabolic factors such as aging, obesity, and traumatic joint injury [53]. Generally, the level of IL-1 family in the synovial fluid, synovial membrane, articular cartilage, and subchondral bone is elevated in the synovial joint of patients with OA [54]. After IL-1 family binds onto their receptors, it manifests the progressive degeneration of articular cartilage by the expression of other cytokines, chemokines, adhesion molecules, inflammatory mediators, and cartilage-degrading enzymes through the phosphorylation of cellular signaling transcriptional factors such as the NF*κ*B and MAPKs [54]. As shown in Figure 7, acteoside not only reduced the phosphorylation of ERK1/2, p38, and JNK but also inhibited the phosphorylation of NF*κ*B in the primary rat chondrocytes treated with IL-1*β*. Moreover, Figure 8 shows that acteoside inhibited the translocation of NF*κ*B from the cytosol to nucleus in the primary rat chondrocytes treated with IL-1*β*. Therefore, our results consistently indicate that acteoside counteract the IL-1*β*-induced catabolic effects such as the expression of cartilage-degrading enzymes and the production of proinflammatory cytokines and inflammatory mediators through the inactivation of cellular signaling pathways such as MAPK and NF*κ*B in the primary rat chondrocytes. Recently, similar with our study, Qiao et al. have reported that acteoside inhibits inflammatory response in OA-induced animals [55]. They showed the suppression of inflammatory cytokines through the inactivation of the JAK/STAT signaling pathway in the synovial tissue of DMM-induced OA animals that were administered intraperitoneal injection of acteoside [55]. However, to estimate the effectiveness of acteoside as an OA preventive supplement, acteoside was orally administrated to DMM-induced OA animals in the present study. Thereafter, the alteration of articular cartilage was histologically assessed as shown in Figure 9. Our histological assessment showed that the oral administration of acteoside consistently prevented the progressive degeneration of articular cartilage through the inhibition of proteoglycan loss in DMM-induced OA animals.

## 5. Conclusions

Our findings suggest that acteoside is capable for oral administration and may be used as an effective supplement to prevent or attenuate OA based on the biological safety and anticatabolic effects against proinflammatory cytokines.

## Figures and Tables

**Table 1:** Quantitative PCR primer sequences used in this study.

Gene	Primer sequences	NCBI gene No.
MMP-13	Forward: 5′-GGCAAAAGCCATTTCATGCTCCCA-3′	NM_133530.1
	Reverse: 5′-AGACAGCATCTACTTTGTCGCCA-3′	
MMP-1	Forward: 5′-CAACGCAGATTTAGCCTCCGA-3′	NM_001134530.1
	Reverse: 5′-GAGATGCCCAGGACCACAGT-3′	
MMP-3	Forward: 5′-TCCTACCCATTGCATGGCAGTGAA-3′	NM_133523.3
	Reverse: 5′-GCATGAGCCAAGACCATTCCAGG-3′	
iNOS	Forward: 5′-GCATCGGCAGGATTCAGTGG-3′	NM_012611.3
	Reverse: 5′-TAGCCAGCGTACCGGATGAG-3′	
COX-2	Forward: 5′-CCCTTCCTCCTGTGGCTGAT-3′	NM_017232.3
	Reverse: 5′-CCCAGGTCCTCGCTTCTGAT-3′	
GAPDH	Forward: 5′-TGATGCTGGTGCTGAGTATG-3′	NM_017008.4
	Reverse: 5′-GGATGCAGGGATGATGTTCT-3′	

**Table 2:** Quantitative real-time PCR primer sequences used in this study.

Gene	Primer sequences	NCBI gene No.
MMP-13	Forward: 5′-TTGGCTTAGATGTGACTGGC-3′	NM_133530.1
	Reverse: 5′-CCCTCGAACACTCAAATGGT-3′	
MMP-1	Forward: 5′-CTACCAGCTCATACAGTTTCCC-3′	NM_001134530.1
	Reverse: 5′-CTACAACTTGGGTGAAGACGT-3′	
MMP-3	Forward: 5′-GTCTTGAAAAGGATGTGAAGCAG-3′	NM_133523.3
	Reverse: 5′-CTCGAACACTATGGAGCTGATG-3′	
GAPDH	Forward: 5′-AACCCATCACCATCTTCCAG-3′	NM_017008.4
	Reverse: 5′-CTGGTGCTGAGTATGTCGTG-3′	

## Data Availability

The data used to support the findings of this study are available from the corresponding author upon request.

## Abbreviations

ADAMTs: A disintegrin and metalloproteinase with thrombospondin motifs
*β*-NGF: *β*-Nerve growth factor
CINC: Cytokine-induced neutrophil chemoattractant
CNTF: Ciliary neurotrophic factor
COX-2: Cyclooxygenase-2
CX3CL1: Fractalkine
DMEM/F12: Dulbecco's Modified Eagle's Medium/Nutrient Mixture F-12
DMM: Destabilization of the medial meniscus
DMMB: Dimethylmethylene blue
ECM: Extracellular matrix
FBS: Fetal bovine serum
GAPDH: Glyceraldehyde 3-phosphate dehydrogenase
IACUC: Institutional Animal Care and Use Committee
IL-1*β*: Interleukin-1*β*
iNOS: Inducible nitric oxide synthase
MAPK: Mitogen-activated protein kinase
MCP-1: Monocyte chemoattractant protein-1
mini-ITS: Mini-insulin-transferrin-selenium
MIP: Macrophage inflammatory protein
MMP: Matrix metalloproteinase
MTT: 3-(4,5-dimethylthiazol-2-yl)-2,5-diphenyl tetrazolium bromide
NF*κ*B: Nuclear factor-kappa B
NO: Nitric oxide
OA: Osteoarthritis
PGE_2_: Prostaglandin E_2_
PTGS2: Prostaglandin-endoperoxide synthase 2
qPCR: Quantitative polymerase chain reaction
qRT-PCR: Quantitative real-time polymerase chain reaction
SDS-PAGE: Sodium dodecyl sulfate-polyacrylamide gel electrophoresis.

## References

[1] Di Chen J. S., Zhao W., Wang T., Han L., Hamilton J. L., Im H.-J. (2017). Osteoarthritis: toward a comprehensive understanding of pathological mechanism. *Bone Research*.

[2] Musumeci G., Aiello F. C., Szychlinska M. A., Di Rosa M., Castrogiovanni P., Mobasheri A. (2015). Osteoarthritis in the XXIst century: risk factors and behaviours that influence disease onset and progression. *International Journal of Molecular Sciences*.

[3] Ghouri A., Conaghan P. G. (2020). Prospects for therapies in osteoarthritis. *Calcified Tissue International*.

[4] Klimek B. (1996). 6′-0-Apiosyl-verbascoside in the flowers of mullein (Verbascum species). *Acta Poloniae Pharmaceutica*.

[5] Pardo F., Perich F., Villarroel L., Torres R. (1993). Isolation of verbascoside, an antimicrobial constituent of Buddleja globosa leaves. *Journal of Ethnopharmacology*.

[6] Henn J. G., Steffens L., de Moura Sperotto N. D. (2019). Toxicological evaluation of a standardized hydroethanolic extract from leaves of *Plantago australis* and its major compound, verbascoside. *Journal of Ethnopharmacology*.

[7] Khullar M., Sharma A., Wani A. (2019). Acteoside ameliorates inflammatory responses through NFkB pathway in alcohol induced hepatic damage. *International Immunopharmacology*.

[8] Hwang T. W., Kim D. H., Kim D. B. (2019). Synergistic anticancer effect of acteoside and temozolomide-based glioblastoma chemotherapy. *International Journal of Molecular Medicine*.

[9] Li X., Xie Y., Li K. (2018). Antioxidation and cytoprotection of acteoside and its derivatives: comparison and mechanistic chemistry. *Molecules*.

[10] Li M., Zhou F., Xu T., Song H., Lu B. (2018). Acteoside protects against 6-OHDA-induced dopaminergic neuron damage via Nrf2-ARE signaling pathway. *Food and Chemical Toxicology*.

[11] Santos-Cruz L. F., Ávila-Acevedo J. G., Ortega-Capitaine D. (2012). Verbascoside is not genotoxic in the ST and HB crosses of the *Drosophila* wing spot test, and its constituent, caffeic acid, decreases the spontaneous mutation rate in the ST cross. *Food and Chemical Toxicology*.

[12] Negoro K., Kobayashi S., Takeno K., Uchida K., Baba H. (2008). Effect of osmolarity on glycosaminoglycan production and cell metabolism of articular chondrocyte under three-dimensional culture system. *Clinical and Experimental Rheumatology*.

[13] You J. S., Cho I. A., Kang K. R. (2017). Coumestrol counteracts interleukin-1*β*-induced catabolic effects by suppressing inflammation in primary rat chondrocytes. *Inflammation*.

[14] Pauli C., Whiteside R., Heras F. L. (2012). Comparison of cartilage histopathology assessment systems on human knee joints at all stages of osteoarthritis development. *Osteoarthritis and Cartilage*.

[15] Henson F. M. D., Vincent T. A. (2008). Alterations in the vimentin cytoskeleton in response to single impact load in an in vitro model of cartilage damage in the rat. *BMC Musculoskeletal Disorders*.

[16] Corciulo C., Cronstein B. N. (2019). Signaling of the purinergic system in the joint. *Frontiers in Pharmacology*.

[17] Neogi T. (2013). The epidemiology and impact of pain in osteoarthritis. *Osteoarthritis and Cartilage*.

[18] Hall A. C. (2019). The role of chondrocyte morphology and volume in controlling phenotype-implications for osteoarthritis, cartilage repair, and cartilage engineering. *Current Rheumatology Reports*.

[19] Leong D. J., Hardin J. A., Cobelli N. J., Sun H. B. (2011). Mechanotransduction and cartilage integrity. *Annals of the New York Academy of Sciences*.

[20] Kapoor M., Martel-Pelletier J., Lajeunesse D., Pelletier J. P., Fahmi H. (2011). Role of proinflammatory cytokines in the pathophysiology of osteoarthritis. *Nature Reviews Rheumatology*.

[21] Henrotin Y., Mobasheri A. (2018). Natural products for promoting joint health and managing osteoarthritis. *Current Rheumatology Reports*.

[22] He J., Hu X. P., Zeng Y. (2011). Advanced research on acteoside for chemistry and bioactivities. *Journal of Asian Natural Products Research*.

[23] Xiong L., Mao S., Lu B. (2016). Osmanthus fragrans flower extract and acteoside protect against d-galactose-induced aging in an ICR mouse model. *Journal of Medicinal Food*.

[24] Perucatti A., Genualdo V., Pauciullo A. (2018). Cytogenetic tests reveal no toxicity in lymphocytes of rabbit (Oryctolagus cuniculus, 2n=44) feed in presence of verbascoside and/or lycopene. *Food and Chemical Toxicology*.

[25] Thielen N. G., van der Kraan P. M., van Caam A. P. (2019). TGFbeta/BMP signaling pathway in cartilage homeostasis. *Cell*.

[26] Mehana E.-S. E., Khafaga A. F., El-Blehi S. S. (2019). The role of matrix metalloproteinases in osteoarthritis pathogenesis: an updated review. *Life Sciences*.

[27] Thorson C., Galicia K., Burleson A. (2019). Matrix metalloproteinases and their inhibitors and proteoglycan 4 in patients undergoing total joint arthroplasty. *Clinical and Applied Thrombosis/Hemostasis*.

[28] Chow Y. Y., Chin K.-Y. (2020). The role of inflammation in the pathogenesis of osteoarthritis. *Mediators of Inflammation*.

[29] Sasaki K., Hattori T., Fujisawa T., Takahashi K., Inoue H., Takigawa M. (1998). Nitric oxide mediates interleukin-1-induced gene expression of matrix metalloproteinases and basic fibroblast growth factor in cultured rabbit articular chondrocytes. *Journal of Biochemistry*.

[30] Goggs R., Carter S. D., Schulze-Tanzil G., Shakibaei M., Mobasheri A. (2003). Apoptosis and the loss of chondrocyte survival signals contribute to articular cartilage degradation in osteoarthritis. *Veterinary Journal*.

[31] Rahmati M., Mobasheri A., Mozafari M. (2016). Inflammatory mediators in osteoarthritis: a critical review of the state-of-the-art, current prospects, and future challenges. *Bone*.

[32] Gouze J.-N., Gouze E., Popp M. P. (2006). Exogenous glucosamine globally protects chondrocytes from the arthritogenic effects of IL-1beta. *Arthritis Research & Therapy*.

[33] Zhang R. X., Ren K., Dubner R. (2013). Osteoarthritis pain mechanisms: basic studies in animal models. *Osteoarthritis and Cartilage*.

[34] Im H. J., Kim J. S., Li X. (2010). Alteration of sensory neurons and spinal response to an experimental osteoarthritis pain model. *Arthritis and Rheumatism*.

[35] Wu F., Zhang R., Shen X., Lao L. (2014). Preliminary study on pain reduction of monosodium iodoacetate-induced knee osteoarthritis in rats by carbon dioxide laser moxibustion. *Evidence-based Complementary and Alternative Medicine*.

[36] Sims N. A., Walsh N. C. (2010). GP130 cytokines and bone remodelling in health and disease. *BMB Reports*.

[37] Montagnoli C., Tiribuzi R., Crispoltoni L. (2017). *β*-NGF and *β*-NGF receptor upregulation in blood and synovial fluid in osteoarthritis. *Biological Chemistry*.

[38] Miyagi M., Ishikawa T., Kamoda H. (2017). Efficacy of nerve growth factor antibody in a knee osteoarthritis pain model in mice. *BMC Musculoskeletal Disorders*.

[39] Berenbaum F. (2019). Targeting nerve growth factor to relieve pain from osteoarthritis: what can we expect?. *Joint, Bone, Spine*.

[40] Miller R. E., Block J. A., Malfait A. M. (2017). Nerve growth factor blockade for the management of osteoarthritis pain: what can we learn from clinical trials and preclinical models?. *Current Opinion in Rheumatology*.

[41] Sandell L. J., Xing X., Franz C., Davies S., Chang L. W., Patra D. (2008). Exuberant expression of chemokine genes by adult human articular chondrocytes in response to IL-1beta. *Osteoarthritis and Cartilage*.

[42] Cho I. A., Kim T. H., Lim H. (2019). Formononetin antagonizes the interleukin-1*β*-induced catabolic effects through suppressing inflammation in primary rat chondrocytes. *Inflammation*.

[43] Hou S. M., Hou C. H., Liu J. F. (2017). CX3CL1 promotes MMP-3 production via the CX3CR1, c-Raf, MEK, ERK, and NF-*κ*B signaling pathway in osteoarthritis synovial fibroblasts. *Arthritis Research & Therapy*.

[44] Zhao L., Wang Q., Zhang C., Huang C. (2017). Genome-wide DNA methylation analysis of articular chondrocytes identifies TRAF1, CTGF, and CX3CL1 genes as hypomethylated in osteoarthritis. *Clinical Rheumatology*.

[45] Xu Y.-k., Ke Y., Wang B., Lin J.-h. (2015). The role of MCP-1-CCR2 ligand-receptor axis in chondrocyte degradation and disease progress in knee osteoarthritis. *Biological Research*.

[46] Alaaeddine N., Antoniou J., Moussa M. (2015). The chemokine CCL20 induces proinflammatory and matrix degradative responses in cartilage. *Inflammation Research*.

[47] Hamrick M. W., Herberg S., Arounleut P. (2010). The adipokine leptin increases skeletal muscle mass and significantly alters skeletal muscle miRNA expression profile in aged mice. *Biochemical and Biophysical Research Communications*.

[48] Ku J. H., Lee C. K., Joo B. S. (2009). Correlation of synovial fluid leptin concentrations with the severity of osteoarthritis. *Clinical Rheumatology*.

[49] Yan M., Zhang J., Yang H., Sun Y. (2018). The role of leptin in osteoarthritis. *Medicine (Baltimore)*.

[50] Kroon F. P. B., Veenbrink A. I., de Mutsert R. (2019). The role of leptin and adiponectin as mediators in the relationship between adiposity and hand and knee osteoarthritis. *Osteoarthritis and Cartilage*.

[51] Gao Y. H., Zhao C. W., Liu B. (2020). An update on the association between metabolic syndrome and osteoarthritis and on the potential role of leptin in osteoarthritis. *Cytokine*.

[52] Acuna A. J., Samuel L. T., Jeong S. H., Emara A. K., Kamath A. F. (2020). Viscosupplementation for hip osteoarthritis: does systematic review of patient-reported outcome measures support use?. *Journal of Orthopaedics*.

[53] Sokolove J., Lepus C. M. (2013). Role of inflammation in the pathogenesis of osteoarthritis: latest findings and interpretations. *Therapeutic Advances in Musculoskeletal Disease*.

[54] Wojdasiewicz P., Poniatowski Ł. A., Szukiewicz D. (2014). The role of inflammatory and anti-inflammatory cytokines in the pathogenesis of osteoarthritis. *Mediators of Inflammation*.

[55] Qiao Z., Tang J., Wu W., Tang J., Liu M. (2019). Acteoside inhibits inflammatory response via JAK/STAT signaling pathway in osteoarthritic rats. *BMC Complementary and Alternative Medicine*.

